# Impact of High-Density Lipoproteins on Sepsis

**DOI:** 10.3390/ijms232112965

**Published:** 2022-10-26

**Authors:** Bart De Geest, Mudit Mishra

**Affiliations:** 1 Centre for Molecular and Vascular Biology, Catholic University of Leuven, 3000 Leuven, Belgium; 2 Department of Cardiothoracic Surgery, University Medical Centre Utrecht, 3508 GA Utrecht, The Netherlands

**Keywords:** sepsis, high-density lipoproteins, pathogen-associated lipids, apolipoprotein A-I, phospholipid transfer protein, cholesterol ester transfer protein, reverse lipopolysaccharide transport

## Abstract

Sepsis is a life-threatening organ dysfunction caused by a dysregulated host response to infection. Here, we review the impact of high-density lipoproteins (HDL) on sepsis from the perspective of biochemistry and pathophysiology, epidemiological research, and intervention studies in animals. Pathogen lipid moieties are major ligands for innate immunity receptors, such as toll-like receptors. The binding of pathogen-associated lipids to lipoproteins leads to sequestration, neutralization, and inactivation of their pro-inflammatory effects. Lipoproteins constitute an arm of the innate immune system. Pathogen-associated lipids can be removed from the body via the reverse lipopolysaccharide transport pathway in which HDL play a key role. Independent of the capacity for sequestration, the direct anti-inflammatory effects of HDL may counteract the development of sepsis. Mendelian randomization research using genetic variants associated with HDL cholesterol as an instrumental variable was consistent with a probable causal relationship between increased HDL cholesterol levels and decreased risk of infectious hospitalizations. Low HDL cholesterol independently predicts an adverse prognosis in sepsis both in observational epidemiology and in Mendelian randomization studies. Several HDL-associated enzymes, including phospholipid transfer protein (PLTP) and cholesterol ester transfer protein (CETP), undergo profound changes during sepsis. Potential HDL-directed interventions for treatment of sepsis include apolipoprotein A-I-based therapies, recombinant PLTP, and CETP inhibition.

## 1. Definition of Sepsis and Overview

Sepsis is a life-threatening organ dysfunction caused by a dysregulated host response to infection [1]. The dysregulated inflammatory response to infection and the altered metabolic state induce tissue injury, organ failure, and frequently death. Sepsis is associated with a mortality of approximately 30% [2] and is a major cause of in-hospital mortality [3]. The concept of dysregulation not only pertains to hyperinflammation but also to immunosuppression [4], since excessive inflammation and immunoparalysis can co-exist from the inception of sepsis [4]. From an operational point of view, sepsis is defined by sepsis-3-criteria, which include a suspected infection by the treating physician and an increase in the sequential organ failure assessment (SOFA) score of at least two points [1,5,6]. The incidence of sepsis is rising, which is related to the ageing of the population and the increased prevalence of patients on immunosuppressive drugs.

This review has been structured in 10 sections and aims to provide insights into the impact of HDL on sepsis from distinct fields and viewpoints. After discussing the key role of pathogen-associated lipids and innate immunity in sepsis (Section 2), we examine the sequestration of pathogen-associated lipids by lipoproteins and the reverse lipopolysaccharide transport pathway (Section 3), the elimination of lipopolysaccharide and the function of parenchymal liver cells and sinusoidal liver cells in this process (Section 4), and the role of lipoproteins in the elimination of lipopolysaccharide (Section 5). HDL delivers cholesterol to the adrenal gland for adrenal corticosteroid production, which represents a critical regulatory process to maintain homeostasis in sepsis (Section 6). HDL cholesterol levels are an independent prognostic factor in sepsis and Mendelian randomisation studies clearly suggest that the observed associations likely represent a causal relationship (Section 7). Apolipoprotein A-I is the main apolipoprotein of HDL and apolipoprotein A-I-based strategies in the treatment of sepsis are presented in Section 8, whereas the modulation of HDL metabolism by targeting HDL-associated enzymes for the treatment of sepsis is discussed in Section 9. Finally, we present conclusions and future perspectives in Section 10.

## 2. Pathogen-Associated Lipids and Innate Immunity

Pathogen lipid moieties, such as lipopolysaccharide in Gram-negative bacteria, lipoteichoic acid in Gram-positive bacteria, and phospholipomannan in the fungus *Candida albicans* are major ligands for innate immunity receptors, such as toll-like receptors [7]. Toll-like receptors are categorized as pattern recognition receptors and sense pathogen-associated molecular patterns [8]. They are typically expressed in sentinel cells, including antigen-presenting cells. Toll-like receptors can stimulate the immune system. The activation of toll-like receptor signalling turns on nuclear factor (NF)-κB, which triggers the production of pro-inflammatory cytokines and initiates antigen presentation functions. The secretion of pro-inflammatory cytokines by innate immune myeloid cells engages lymphocytes to mount an adaptive, antigen-specific immune response that ultimately is directed at the eradication of the invading microbes.

An excessive, dysregulated host response underlies sepsis. Pathogen-associated lipids, including lipopolysaccharide from Gram-negative bacteria and lipoteichoic acid from Gram-positive bacterial cell walls, may be the principal pathogenetic drivers of the change from a straightforward, manageable infection to sepsis [9]. Lipopolysaccharide-binding protein (LBP) is an acute-phase protein that is accountable for the binding and transport of lipopolysaccharide in the circulation [10]. The interaction of lipopolysaccharide with LBP leads to the binding of lipopolysaccharide to CD14 on macrophages. LBP and CD14 catalyse lipopolysaccharide transfer to the toll-like receptor 4/MD2 complex [11]. This results in the activation of an inflammatory response through NF-κB and mitogen-activated protein kinase (MAPK) signalling pathways [12].

## 3. Sequestration of Pathogen-Associated Lipids by Lipoproteins and Reverse Lipopolysaccharide Transport Pathway

Binding of lipopolysaccharide and other toxic pathogen lipids to lipoproteins of different classes is of paramount importance because this process of sequestration results in the neutralization and inactivation of the pro-inflammatory effects of these toxic lipids [13,14,15,16,17]. The sequestration of lipopolysaccharide into lipoproteins will prevent interaction with toll-like receptor 4 and will abrogate hyperinflammation. Since pathogen lipid moieties are key molecules in the pathogenesis of sepsis, it is, therefore, no surprise that plasma levels circulating lipoproteins may modify the course of infection and sepsis by the increased neutralisation of the effect of these pathogen-associated lipids. More than 90% of lipopolysaccharide in human plasma has been demonstrated to be bound to lipoproteins [18]. The affinity for lipopolysaccharide is highest for high-density lipoproteins (HDL), medium for low-density lipoproteins (LDL), and low for very low-density lipoproteins (VLDL) [18]. Affinity is determined by the phospholipid component of lipoproteins [19], which is highest in HDL [20]. The saturation capacity of lipoproteins for lipopolysaccharide is in excess of 200 µg/mL [18]. HDL particles can be considered to constitute bug scavengers [21]. Lipopolysaccharide bound to HDL does not stimulate cytokine production in vitro or in vivo [22]. HDL has the highest affinity not only for lipopolysaccharide but also for lipoteichoic acid [14,18]. An inverse correlation exists between HDL cholesterol levels and LDL cholesterol levels on the one hand and circulating levels of tumour necrosis factor-α, interleukin-6, and interleukin-10 on the other hand in patients with sepsis [23]. This is compatible with effective neutralisation of pathogen-associated lipids by these lipoproteins in vivo.

**Figure 1:**
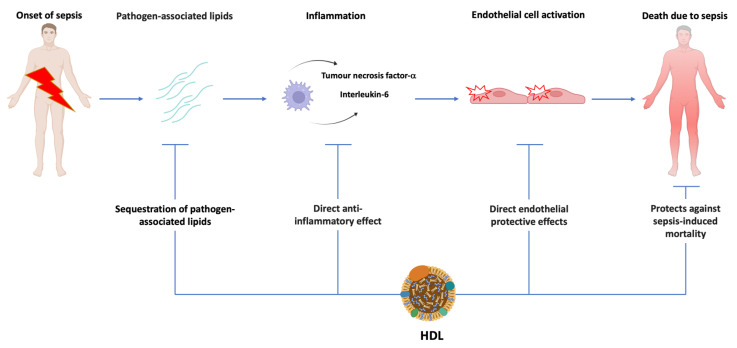
**General overview of the mechanisms that contribute to the protective effects of HDL in sepsis.** HDL have the potential of sequestration and neutralization of pathogen-associated lipids, exert direct anti-inflammatory effects, and can also counteract activation of the endothelium induced by cytokines. Increased HDL cholesterol predicts a lower mortality in patients with sepsis.

The impact of HDL on sepsis is not solely mediated via neutralisation of pathogen-associated lipids (Figure 1). HDL are multimolecular platforms and have pleiotropic properties [24]. HDL particles predominantly contain apolipoprotein A-I, cholesterol, and phospholipids, but biochemical heterogeneity of these particles is determined by a changeable presence of one or more representatives of at least 250 proteins, 300 lipid species, and 20 micro RNAs [25,26]. These pleiotropic properties and, in particular, the anti-inflammatory effects of HDL are relevant in sepsis [24]. HDL upregulates the activating transcription factor 3 [27]. Activating transcription factor 3 is a negative regulator in the toll-like receptor 4 signalling pathway [28]. One of the mechanisms of repression is the negative regulation of NF-κB by activating transcription factor 3 via direct interaction with p65 [29]. The repression of toll-like receptor signalling results in a decrease in downstream proinflammatory cytokines, such as tumour necrosis factor-α, interleukin-6, and interleukin-12p40.

As will be further discussed and highlighted in this review, the neutralisation and subsequent elimination of pathogen-associated lipids by lipoproteins has a major impact on the course of sepsis [18,30]. In this respect, the existence of a reverse lipopolysaccharide transport pathway was postulated more than 10 years ago [31], which has similarities with reverse cholesterol transport from the periphery to the liver for excretion into bile [32,33]. In both pathways, HDL play a key role.

## 4. Elimination of Lipopolysaccharide

Since elevated lipopolysaccharide levels are a predictor of adverse outcomes in patients with severe sepsis [34], understanding the mechanisms of lipopolysaccharide clearance is highly relevant. In the elimination of lipopolysaccharide, a distinction should be made between lipopolysaccharide sequestered into lipoproteins and lipopolysaccharide not bound to lipoproteins. In this section, the focus is on the clearance of lipopolysaccharide that is not bound to lipoproteins and can, therefore, cause a dysregulated host immune response. The removal of lipopolysaccharide sequestered into lipoproteins will be discussed in the next section. As will be discussed there, Topchiy et al. [17] demonstrated a key role of LDL receptor expression in parenchymal liver cells in the removal of lipopolysaccharide.

The principal mechanism of the removal of lipopolysaccharide from the bloodstream is uptake by the liver [35]. Parenchymal cells constitute 66% of liver cells, whereas liver sinusoidal cells, containing both Kupffer cells and liver sinusoidal endothelial cells, comprise 33% of liver cells [36]. The ratio of liver sinusoidal endothelial cells to Kupffer cells is approximately 3.5 to 1 [36]. Kupffer cells are generally responsible for uptake and clearance of larger particles, such as bacteria or cells, by phagocytosis [19]. In contrast, liver sinusoidal endothelial cells can endocytose particles up to 230 nm [36,37,38]. Lipopolysaccharide has a size of 15–50 nm [39].

Both parenchymal liver cells and sinusoidal cells contribute to the removal of lipopolysaccharide. Free lipopolysaccharide in the bloodstream can be eliminated by Kupffer cells and is inactivated in these cells following deacylation, induced by the enzyme acyloxyacyl hydrolase [40]. However, the Kupffer-mediated uptake and modification of lipopolysaccharide happens slowly, typically over hours [41]. Since lipopolysaccharide is eliminated more rapidly from the blood circulation, other mechanisms must be involved [19]. In murine experiments, Yao et al. [19] observed that lipopolysaccharide quickly disappears from the circulation with a half-life of 2–4 min in mice and that the liver removes approximately 75% of lipopolysaccharide from the circulation. The authors further noticed that about three quarters of fluor-tagged lipopolysaccharide in liver was associated with liver sinusoidal endothelial cells and only approximately one quarter with Kupffer cells [19]. Remarkably, the authors did not investigate the role of parenchymal liver cells in the clearance of lipopolysaccharide. Nevertheless, there is convincing experimental evidence that parenchymal liver cells are important for the clearance of lipopolysaccharide.

Experiments in mice in which toll-like receptor 4 was specifically deleted from hepatocytes, i.e., parenchymal liver cells, demonstrated that toll-like receptor 4 expression in hepatocytes is critical for the clearance of lipopolysaccharide, both in a model of polymicrobial sepsis induced by caecal ligation and puncture and in a model of endotoxemia [42]. The same investigation demonstrated that uptake in sinusoidal cells occurs in a toll-like receptor 4-independent manner [42]. The model of caecal ligation and puncture is considered to constitute a representative model of sepsis that is clinically seen in patients with polymicrobial intraperitoneal infection secondary to faecal contamination [43]. However, this model is relatively difficult to standardize [43]. Importantly, under conditions of antibiotic administration in the model of caecal ligation and puncture, a key role of lipopolysaccharide clearance by hepatocytes was demonstrated in limiting sepsis-induced inflammation and organ injury [42]. Specifically, in animals with a hepatocyte-specific deletion of toll-like receptor 4 expression, increased circulating lipopolysaccharide levels, induced by a reduction in lipopolysaccharide clearance in hepatocytes, stimulated toll-like receptors on myeloid cells [42]. This resulted in increased production of inflammatory cytokines, which subsequently produces end-organ damage in sepsis [42]. The lipopolysaccharide-induced internalization of toll-like receptor 4 is governed by the glycosylphosphatidylinositol-anchored protein CD14 [44,45]. Toll-like receptor 4 is the signalling but is not the main lipopolysaccharide uptake receptor [46]. The uptake of lipopolysaccharide into hepatocytes in vivo is CD14-dependent, but other receptors may be involved [47]. Overall, these experiments provided convincing evidence for a key role of hepatocytes in the removal of lipopolysaccharide [42].

In general, lipopolysaccharide clearance is of paramount importance and the capacity to neutralize and/or clear lipopolysaccharide may be highly different between distinct species. The clearance capacity of lipopolysaccharide in mice is tremendous [19]. The dose of lipopolysaccharide required to induce an interleukin-6 plasma concentration of 1000 pg/mL 2 h after injection was 2 ng/kg of body weight in humans and 500 ng/kg in mice [48].

Overall, different experimental approaches to evaluate lipopolysaccharide clearance by the parenchymal and sinusoidal cells of the liver do not yield concordant results at first glance. The apparent discrepancy in experiment results on the role of hepatocytes, Kupffer cells, and liver sinusoidal cells in lipopolysaccharide clearance may be explained by substantial differences in the experimental models. First of all, these differences relate to variations in the amount of administered lipopolysaccharide and to dissimilarities in the purity of lipopolysaccharide in investigations evaluating exogenous lipopolysaccharide administration. There is also heterogeneity in the structure of bacterial lipopolysaccharides [49]. Secondly, distinctions arise as a result of whether or not antibiotics are administered in the model of polymicrobial sepsis induced by caecal ligation. Results in this model are also dependent on the amount of cecum ligated and the needle puncture size [43]. Thirdly, murine experiments are highly dependent on the specific strain of mice that is investigated [50,51]. Fourthly, differentiating the relative contribution of Kupffer cells and liver sinusoidal endothelial cells is not straightforward, and the role of the latter cell type is often neglected.

## 5. Role of Lipoproteins in Lipopolysaccharide Elimination

### 5.1. Reverse Lipopolysaccharide Transport

The bulk of lipopolysaccharide cannot be metabolized in the body and must be eliminated. Pathogen-associated lipids are sequestered within HDL, LDL, and VLDL and effectively neutralized. Lipoproteins could be considered to constitute an arm of the innate immune system [52]. The dissociation rate of lipopolysaccharide from lipopolysaccharide–lipoprotein complexes is significantly slower than the clearance rate of lipoproteins, and thus lipoprotein metabolism is crucial for elimination of lipopolysaccharide [19]. Lipopolysaccharide can be removed from the body via the reverse lipopolysaccharide transport pathway [31], which has significant similarities with reverse cholesterol transport [32,33]. The reverse lipopolysaccharide transport pathway involves lipopolysaccharide disaggregation and its binding to lipoproteins, the transport of lipoprotein-bound lipopolysaccharide to the liver, the uptake of lipoprotein-bound lipopolysaccharide into the liver, and the excretion of lipopolysaccharide into bile [31]. In addition, circulating lipoproteins, reverse lipopolysaccharide transport involves proteins of the lipid transfer/lipopolysaccharide-binding protein gene family. In addition to LBP, the lipid transfer/lipopolysaccharide-binding protein gene family includes the bactericidal permeability increasing protein, phospholipid transfer protein (PLTP), and cholesterol ester transfer protein (CETP) [53]. All these proteins are involved in innate immunity and two of these proteins (CETP, PLTP) play a key role in HDL metabolism and constitute targets for treatment of sepsis, as will be discussed in Section 9. As already mentioned, LBP is an acute-phase protein and can, together with CD14, catalyse the transfer of lipopolysaccharide to the toll-like receptor-4/MD2 complex, inducing the dimerization of the toll-like receptor-4//MD2 complex, contributing to monocyte activation and a pro-inflammatory cascade [11]. Lipopolysaccharide-bound LBP can also transfer lipopolysaccharide to lipoproteins, resulting in endotoxin inactivation and detoxification. The delivery of lipopolysaccharide to HDL by LBP results in the attenuation of the immune response to infection, whereas delivery of LPS by LBP to macrophage receptors initiates signal transduction pathways, resulting in the enhanced production of proinflammatory cytokines [13]. Lipopolysaccharide is redistributed among the major lipoprotein subclasses in a process involving LBP and PLTP [13]. Both proteins transfer lipopolysaccharide from HDL to predominantly LDL in a time- and dose-dependent manner [13]. As reported by Dusuel et al. [54], CETP lacks lipopolysaccharide transfer activity. This transfer from HDL to apolipoprotein B-containing lipoproteins is important, since direct transport of lipopolysaccharide to parenchymal liver cells by HDL is likely limited.

### 5.2. Role of LDL Receptor in Lipopolysaccharide Clearance

Lipopolysaccharide that is bound to LDL can be cleared from the circulation by hepatocytes via the LDL receptor. There is indeed substantial experimental evidence that the LDL receptor plays a crucial role in lipoprotein-bound lipopolysaccharide clearance in vivo [17]. Topchiy et al. [17]. demonstrated that that clearance of injected lipopolysaccharide from the blood was markedly lower in LDL receptor-deficient mice than in wild-type control mice. Moreover, the hepatic uptake of lipopolysaccharide was decreased in LDL receptor-deficient mice, clearly indicating the role of the LDL receptor in the liver uptake of lipopolysaccharide. In vitro experiments with primary hepatocytes isolated from wild-type and LDL receptor-deficient mice showed that the uptake and internalization of lipopolysaccharide in the presence of serum was substantially reduced in hepatocytes lacking LDL receptor expression [17]. Importantly, the impact of sepsis on LDL receptor expression in the liver appears to be species dependent. In humans, interleukin-1 and tumour necrosis factor-α increase LDL receptor activity [55]. In contrast, LDL clearance from the circulation is prominently reduced in rats by lipopolysaccharide, which is secondary to the posttranscriptional downregulation of the LDL receptor during inflammation [17,55]. As a result of this and because of profound differences in lipoprotein metabolism between different species [32], alterations of lipoprotein levels during sepsis in humans are distinct, compared to other species.

### 5.3. Impact of Proprotein Convertase Subtilisin/Kexin Type 9

The bidirectional relationship between sepsis and lipoprotein metabolism is further underscored by the effect of sepsis on the levels of proprotein convertase subtilisin/kexin type 9 (PCSK9). PCSK9 is an enzyme that interferes with the recycling of the LDL receptor to the plasma membrane and induces increased lysosomal degradation of the LDL receptor [56]. PCSK9 is a cholesterol-regulated gene and strongly upregulated by sterol regulatory-element binding protein (SREBP)-1a and SREBP-2 [57]. However, inflammation may also induce increased expression of PCSK9 [58]. The concentrations of PCSK9 increase transiently over time in critically ill patients both with sepsis and without sepsis, with very similar profiles over 14 days [59]. Using flow cytometry, it was demonstrated that PCSK9 downregulates LDL receptor-mediated uptake of lipoteichoic acid and lipopolysaccharide by HepG2 hepatocytes through a mechanism that involves LDL particles [59]. Interestingly, increased levels of PCSK9 are a prognostic biomarker in sepsis [60]. Moreover, septic shock patients with PCSK9 loss-of-function mutations were characterized by a decrease in inflammatory cytokine response and by improved survival [61,62]. An attenuation of inflammatory cytokines response was also observed following lipopolysaccharide administration in healthy volunteers with PCSK9 loss-of-function genetic variants. A whole series of experiments substantiates the thesis that reduced PCSK9 function and the consequent increase in LDL receptor expression in hepatocytes is associated with increased pathogen lipid clearance via the LDL receptor, a diminished inflammatory response, and improved survival following septic shock [61,62]. Finally, decreased PCSK9 function increases lipoteichoic acid clearance and improves outcomes in patients with Gram-positive septic shock [63]. 

PCSK9 is also involved in the degradation of the VLDL receptor [64] and adipose tissue may play a secondary role in the removal of lipopolysaccharide via VLDL receptor uptake [65], which may explain why sepsis prognosis is better in obese patients [7,66]. PCSK9 may also exert effects independent of its effects on cholesterol metabolism. PCSK9 may enhance the secretion of inflammatory factors in macrophages through modulating the expression of toll-like receptor 4/NF-κB [67]. PCSK9 overexpression upregulates toll-like receptor 4 expression and phosphorylation of the inhibitory protein IκBα, leading to increased p-IκBα levels, IkBα degradation, and NF-κB nuclear translocation in macrophages, whereas PCSK9 knockdown had the opposite effects [67]. 

Although the majority of studies confirm that PCSK9 is a prognostic factor in sepsis [7], a number of studies are not concordant [68,69]. This could reflect heterogeneity of patients with sepsis but could also indicate that interpretation of literature may be hampered by publication bias.

### 5.4. Direct Output of Lipopolysaccharide to the Liver by HDL

The question arises whether there is direct output of lipopolysaccharide to the liver by HDL or whether there is only indirect output after transfer to apolipoprotein B-containing lipoproteins that are eliminated via the LDL receptor. Yao et al. [19] demonstrated that fluor-tagged lipopolysaccharide–HDL complexes localized to livers sinusoidal endothelial cells at 3 min after infusion in mice. They speculated that the receptor, which could facilitate the uptake of lipopolysaccharide into liver sinusoidal endothelial cells, is scavenger receptor class B, type I (SR-BI). SR-BI is abundantly expressed in liver sinusoidal endothelial cells [70]. If direct output of lipopolysaccharide from HDL indeed occurs via liver sinusoidal endothelial cells, this raises the issue of how lipopolysaccharide is excreted into bile. One might postulate a transcytosis mechanism involving endocytosis, transport across liver sinusoidal endothelial cells, exocytosis into the space of Disse, and finally re-uptake by parenchymal liver cells. Whether enzymatic inactivation of lipopolysaccharide may occur in liver sinusoidal endothelial cells is unclear.

As already pointed out, there is a bidirectional relationship between cholesterol metabolism and sepsis. Another dimension of this is that cholesterol is a principal component of lipid rafts and caveolae that play a critical role in organization of signalling by toll-like receptors [71]. Therefore, increased cholesterol content of cell membranes may lead to increased production of cytokines and chemokines and an amplification of inflammation.

## 6. Role of HDL in Adrenal Gland Biology: Impact on Sepsis

The importance of HDL in sepsis is also underscored by its role in adrenal corticosteroid production. The activation of the hypothalamic–pituitary–adrenal axis in sepsis results in adrenocortical glucocorticoid production, which represents a critical regulatory process to maintain homeostasis [72]. SR-BI-deficient mice are characterized by deficient HDL metabolism, impaired production of corticosteroids in the adrenals, and female infertility [73,74]. Endotoxemia and polymicrobial sepsis result in higher mortality in SR-BI-deficient mice [75,76,77]. SR-BI-deficient mice were characterized by a deficient inducible glucocorticoid synthesis in response to lipopolysaccharide or to bacterial infection or to stress [77]. Insufficient glucocorticoid production in SR-BI deficient mice was caused by deficient HDL-mediated cholesterol delivery to the adrenals, leading to primary adrenal malfunction [77]. Corticosterone supplementation reduced the sensitivity of SR-BI-deficient animals to lipopolysaccharide. Consistent with the postulated role of SR-BI in lipopolysaccharide clearance discussed in the previous section, the plasma clearance of lipopolysaccharide into the liver was reduced in SR-BI-deficient mice, compared to wild-type mice [76,77]. In aggregate, these data point to the importance of a physiological HDL metabolism to mitigate sepsis.

## 7. Lipoprotein Levels as Prognostic Factor in Sepsis

**Figure 2:**
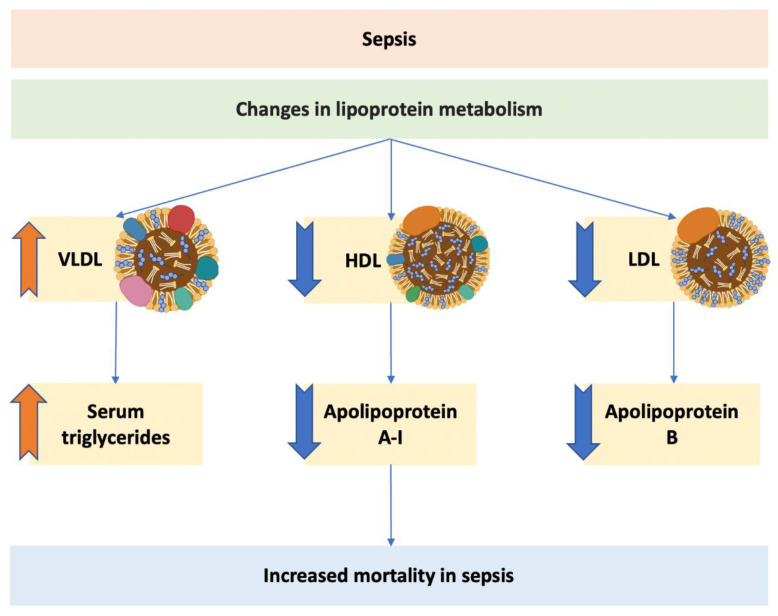
**Bidirectional relationship between sepsis and lipoprotein metabolism**. The decrease in HDL is not only a consequence of sepsis but also an aggravating factor, leading to more severe sepsis and increased sepsis-induced mortality.

Total cholesterol levels are decreased in patients with sepsis, which reflects a reduction in cholesteryl esters and not of non-esterified cholesterol [78]. Serum cholesterol levels can decrease by 50% in patients with severe sepsis [16]. In humans, serum triglyceride and VLDL cholesterol levels are elevated in sepsis, whereas HDL cholesterol and LDL cholesterol levels are reduced (Figure 2) [79]. The increase in triglycerides is explained by increased lipolysis in adipose tissue, leading to an increased supply of free fatty acids and increased lipogenesis and triglyceride synthesis in the liver [23,80]. The infusion of lipopolysaccharide in humans induced an increased phosphorylation of hormone sensitive lipase at Ser^650^, which activates the enzyme and increases lipolysis in adipose tissue [81]. The global impact of sepsis on fatty acid metabolism is complex and a manifestation of a reprogramming of basic metabolic pathways that occurs in sepsis [82]. The decrease in HDL cholesterol in sepsis has often been explained by the displacement of apolipoprotein A-I by the acute phase protein serum amyloid A [83]. This displacement subsequently leads to an increased clearance of apolipoprotein A-I by the kidney [84]. However, this mechanism is uncertain. In this respect, the results of a study by Tanaka et al. [85], which investigated the differences in lipoprotein levels between sepsis and trauma patients, should be carefully considered. In this prospective, observational, single-centred study, 75 consecutive patients (50 sepsis patients and 25 trauma patients) that were admitted to a surgical intensive care unit were included. Both groups did not differ in the Simplified Acute Physiology Score (SAPS) II score, which is used to measure the severity of disease for patients admitted to intensive care units. Evaluation of the lipoprotein profile demonstrated that only HDL cholesterol differed between both groups (median 0.33 mmol/L in sepsis patients versus 0.99 mmol/L in trauma patients). In contrast, no differences were observed in LDL cholesterol levels and triglyceride levels between both groups. Importantly, both sepsis and trauma are characterized by intense systemic inflammation. Unfortunately, serum amyloid A levels were not reported in this paper [85]. Nevertheless, the data suggest that the drop of HDL cholesterol levels in sepsis cannot simply be explained by inflammation and by the increase in serum amyloid A levels, since the drop of HDL cholesterol levels is not observed in trauma patients.

Irrespective of quantitative changes of HDL cholesterol, the compositional changes of HDL during acute inflammation have an impact on HDL function. In particular, the anti-inflammatory properties of HDL are affected [83,84]. The antioxidant potential of HDL is also compromised in sepsis, since the arylesterase activity and paraoxonase activity of HDL-associated paraoxonase 1 (PON1) are reduced in patients with sepsis [86,87]. Notwithstanding the potential pathophysiological relevance of qualitative changes of HDL, the parameters that are important in terms of prognostic prediction models are HDL cholesterol and apolipoprotein A-I, as will be discussed.

Low cholesterol levels, mainly reflecting low HDL cholesterol levels, predict increased mortality in sepsis [88]. Barlage et al. [89] demonstrated that total cholesterol, HDL cholesterol, LDL cholesterol, apolipoprotein A-I and apolipoprotein B were significantly lower in non-surviving sepsis patients than in surviving sepsis patients. Furthermore, HDL cholesterol levels and apolipoprotein A-I levels further decreased in non-survivors during the intensive care unit stay in contrast to apolipoprotein B and LDL cholesterol. Multivariable logistic regression analysis demonstrated that apolipoprotein A-I levels are an independent predictor of 30-day-mortality [89]. Decreased serum levels of HDL cholesterol predict a poor prognosis in adult patients with severe sepsis [90] and in children with severe meningococcal sepsis [91]. Cirstea et al. [92] demonstrated that decreased serum HDL cholesterol levels at the time of emergency department admission for suspected sepsis are an independent predictor of multiple-organ dysfunction and death [92]. The discriminative ability of HDL cholesterol was superior to all other clinical variables that are routinely measured at triage in this patient population [92]. 

The independent prognostic ability of HDL cholesterol as a biomarker raises the question of causality from a clinical perspective. Based on samples of the UK Biobank, Trinder et al. [93] demonstrated that increased levels of measured HDL cholesterol and measured LDL cholesterol were associated with reduced risk of infectious disease hospitalizations. Mendelian randomization using genetic variants associated with HDL cholesterol as an instrumental variable was consistent with a probable causal relationship between increased HDL cholesterol levels and decreased risk of infectious hospitalizations [93]. Even more importantly, there was a significant inverse association between a continuous HDL cholesterol polygenic score and 28-day mortality in sepsis patients [93]. In contrast, continuous LDL cholesterol polygenic score and continuous triglyceride polygenic score were not significantly associated with risk of hospitalization for any infectious disease. The use of genetic scores on Mendelian randomization studies decreases the risk of confounding and reverse causation that can plaque observational studies [94]. The assumption that the genetic instruments are not associated with confounding factors is essentially unproven. Specifically, the assumption in a Mendelian randomization setting that the effects of a gene on a distal outcome only act via the intermediate phenotype (*in casu* HDL cholesterol) is difficult to prove. It is still possible that the intermediate phenotype does not completely capture the effect of the genetic variants. Nevertheless, the results of Trinder et al. [93] support the potential causal role of HDL in infectious diseases and suggest that a therapeutic strategy directed at raising HDL cholesterol levels may be clinically beneficial in patients with severe infections and sepsis.

## 8. Apolipoprotein A-I-Based Strategies in the Treatment of Sepsis

As discussed, HDL has the potential to sequester bacterial toxins, such as lipopolysaccharide and lipoteichoic acid and contributes to the elimination of bacterial toxins [18,30]. The sequestration of pathogen-associated lipids counteracts excessive host inflammation during sepsis [13,14,18]. Protective effects may also be mediated by the anti-inflammatory effects of HDL [24,27]. Whereas the discussion in the previous sections provides converging evidence for a critical role of HDL in sepsis, proof of causality at the level of the organism can only be demonstrated by intervention studies.

In a seminal study, Pajkrt et al. [95] demonstrated that reconstituted HDL, administered via intravenous infusion at a dose of 40 mg/kg initiated 3.5 h before a challenge with endotoxin at a dose of 4 ng/kg, decreased the endotoxin-induced release of tumour necrosis factor-α, interleukin-6, and interleukin-8. Moreover, the infusion of reconstituted HDL per se, before the administration of lipopolysaccharide, resulted in the downregulation of CD14 on monocytes. These data in humans support direct anti-inflammatory effects of HDL in humans. Thus, HDL-targeted therapies might be beneficial in sepsis not only via neutralisation of lipopolysaccharide but also via its anti-inflammatory potential.

Experimental evidence in animals supports the view that the impact of HDL on sepsis is not solely mediated by its potential to sequestrate lipopolysaccharide. Increased HDL cholesterol in the circulation of C57BL/6 mice induced by a human *apolipoprotein A-I* gene transfer vector, which induced persistent transgene expression exclusively in hepatocytes, resulted in an increase in plasma adiponectin levels and adiponectin expression in abdominal fat both in control mice and in lipopolysaccharide-treated mice [96]. Additional in vitro studies indicated that the HDL-mediated effects on adiponectin expression in the presence of lipopolysaccharides are predominantly secondary to an effect that is independent of sequestration of lipopolysaccharide. Adiponectin is an adipokine that increases insulin sensitivity [97] and exerts anti-inflammatory effects [98]. Adiponectin suppresses NF-κB binding to the DNA, leading to suppression of NF-κB target genes, such as C- reactive protein, tumour necrosis factor-α, and interleukin-6. Moreover, human *apolipoprotein A-I* gene transfer had profound effects on the expression of hormone sensitive lipase in adipose tissue and potently abrogated the lipopolysaccharide-induced increase in triglycerides [96].

HDL has also been shown to have an impact on toll-like receptor 4 expression and signalling independent of its potential for lipopolysaccharide sequestration [99]. Lung endothelial toll-like receptor 4 expression was prominently reduced 14 days after human *apolipoprotein A-I* gene transfer in mice. Moreover, toll-like receptor 4 signalling following lipopolysaccharide administration was potently attenuated, as evidenced by reduced expression of myeloid differentiation primary response protein (MyD88) and by decreased activity of NF-κB [99]. The thesis that effects of HDL on toll-like receptor 4 signalling were independent of lipopolysaccharide neutralisation was corroborated by specific in vitro experiments. In these in vitro experiments, HDL or apolipoprotein A-I was only added during the pre-incubation period and all supplementations with lipopolysaccharide were performed in the absence of HDL or apo A–I, clearly providing an experimental framework to demonstrate effects on toll-like receptor 4 signalling independent of lipopolysaccharide sequestration [99].

Apolipoprotein A-I_Milano_ is an apolipoprotein A-I mutant that results from an arginine to cysteine mutation at position 173 and was discovered in 1980 in a family from Limone sul Garda in northern Italy [100,101,102]. Pre-treatment with reconstituted HDL containing recombinant apolipoprotein A-I_Milano_ markedly attenuated liver and renal dysfunction and lung injury induced by the injection of lipopolysaccharide in rats [103]. This was accompanied by a drastic suppression of the endotoxin-induced increase in proinflammatory cytokines and adhesion molecules [103].

Recently, the impact of ETC-642, which consists of a 22–amino acid apolipoprotein A-I mimetic peptide complexed with phospholipids, was evaluated in a murine model of sepsis induced by caecal ligation and puncture [104]. Treatment with ETC-642 resulted in increased HDL cholesterol levels and reduced inflammation, as evidenced by lower levels of interleukin-6 and interleukin-10, better kidney function, and improved survival rate [104]. In vitro studies demonstrated that ETC-642 counteracted the ability of the endotoxins lipopolysaccharide and lipoteichoic acid to activate inflammatory pathways via the respective receptors, toll-like receptor 4 and toll-like receptor 2 [104]. Moreover, ETC-642 inhibited the activation of human umbilical vein endothelial cells by lipopolysaccharide, lipoteichoic acid, and tumour necrosis factor-α.

Notwithstanding these encouraging results, no randomized study using reconstituted HDL has been carried out in patients with sepsis. Therefore, the critical proof of efficacy is still lacking.

## 9. Modulation of HDL Metabolism by Targeting HDL-Associated Enzymes for the Treatment of Sepsis: Recombinant LCAT, Recombinant PLTP, and CETP Inhibition

### 9.1. Changes of Enzymes That Have An impact on HDL Metabolism during Sepsis

Several enzymes that modulate HDL metabolism are associated with HDL particles: lecithin-cholesterol acyl transferase (LCAT), PLTP, and CETP [105]. LCAT catalyses the transfer of a fatty acid from the sn-2 position of phospholipids to free cholesterol to form cholesteryl ester and lysolecithin [106]. PLTP is a member of the lipid transfer/lipopolysaccharide-binding protein gene family, which transfers phospholipids and free cholesterol from triglyceride-rich lipoproteins to HDL [53]. CETP is a hydrophobic glycoprotein, which following secretion from the liver, circulates in plasma, predominantly bound to HDL [107]. This protein is a 74 kDa member of the lipid transfer protein/lipopolysaccharide-binding protein (LTP/LBP) gene family [108]. It mediates the bidirectional transfer of triglycerides and cholesteryl esters between apolipoprotein B-containing, triglyceride-rich lipoproteins and HDL particles, whereby HDL is depleted of its cholesterol content and enriched with triglycerides. Subsequent action of hepatic lipase reduces HDL size and increases catabolism of HDL, which is paralleled by a reduction in apolipoprotein A-I concentrations [32]. Endothelial lipase, which is synthesized by endothelial cells in tissues with high metabolic rates and has enzymatic activity on the surface of endothelial cells, contributes to HDL metabolism via the hydrolysis of HDL phospholipids [109]. In a comparison of 53 intensive care unit sepsis and 25 intensive care unit non-sepsis patients, a significant decrease in lecithin-cholesterol acyl transferase (LCAT) activity and LCAT concentration was observed [110]. CETP levels also decrease in patients with sepsis [110,111] and CETP activity is reduced in sepsis [112,113]. In contrast, PLTP and endothelial lipase activity were significantly elevated in sepsis patients, compared to non-sepsis patients [110].

### 9.2. Recombinant LCAT

LCAT deficiency in mice compromises adrenocortical production of glucocorticoids [114] and reduces the lipopolysaccharide-neutralizing capacity of HDL, resulting in increased inflammation [115]. As already stated, the reduction in serum cholesterol in sepsis reflects a reduction in cholesteryl esters and not of non-esterified cholesterol [78]. Since sepsis reduces LCAT concentration and activity, administration of recombinant LCAT [106,116] could be considered for treatment of sepsis. However, this has not been evaluated until now.

### 9.3. Recombinant PLTP

The association of lipopolysaccharide with lipoproteins neutralizes its endotoxic properties. Intravascular redistribution of lipopolysaccharide from the plasma lipoprotein-free fraction towards circulating lipoproteins was delayed in PLTP-deficient mice [117]. Correspondingly, plasma concentrations of pro-inflammatory cytokines were significantly lower in wild-type mice than in PLTP-deficient mice [117]. Finally, lipopolysaccharide-induced mortality was significantly higher in PLTP-deficient mice than in wild-type mice [117].

The role of PLTP has been further corroborated by the application of recombinant human PLTP [53]. In a model of polymicrobial infection induced by caecal ligation and puncture, recombinant PLTP was shown to disaggregate lipopolysaccharide from the bacterial wall in both PLTP-deficient mice and wild-type mice and to neutralize lipopolysaccharide by transfer to lipoprotein carriers, leading to liver uptake and excretion into bile [53]. This led to a decrease in proinflammatory cytokines and to a reduction in mortality. Remarkably, recombinant human PLTP was also demonstrated to exert direct antibacterial effects, which prevented the growth of Gram-negative bacteria but not Gram-positive bacteria [53]. 

### 9.4. CETP Inhibition

As discussed in Section 9.1, CETP expression and plasma CETP activity are inhibited by lipopolysaccharide [110,111]. This decline may be beneficial since reduced CETP activity may mitigate the sepsis-induced decrease in HDL. Better preservation of HDL levels will lead to improved HDL-mediated sequestration of pathogen associated-lipids. CETP is present in rabbits and humans but absent in rats and mice [32]. A rare missense variant in *CETP* (rs1800777, p.Arg468Gln) was strongly associated with increased CETP mass, lower HDL cholesterol levels, augmented risk of clinically significant sepsis-associated acute kidney injury, and decreased survival in sepsis [118,119]. More recently, a fixed-effect meta-analysis of seven cohorts found that this CETP gain-of-function variant (rs1800777, p.Arg468Gln) was significantly associated with an elevated risk of acute sepsis mortality [113]. Furthermore, a genetic score for decreased CETP function was associated with significantly decreased risk of acute sepsis mortality in the UK Biobank study [113]. The effect of CETP inhibition with anacetrapib 24 h after caecal ligation and puncture was evaluated in APOE*3-Leiden.CETP mice. This treatment resulted in lower CETP activity, higher HDL cholesterol levels, and higher apolipoprotein-A-I levels, compared with mice that received placebo. Intervention with anacetrapib improved kidney function in septic mice and resulted in decreased mortality at 72 h [113]. In APOE*3-Leiden mice, which do not express the CETP transgene, no effect of anacetrapib was observed, indicating that the impact of ancetrapib in APOE*3-Leiden mice is indeed secondary to the inhibition of CETP and not to off-targets effects of anacetrapib. Nevertheless, these results should be contrasted with the studies of Cazita et al. [120] and of Venancio et al. [35]. Cazita et al. [120] demonstrated that administration of lipopolysaccharide derived from *E. Coli* resulted in significantly lower concentrations of tumour necrosis factor-α and interleukin-6 in human CETP transgenic mice, compared to wild-type mice. Remarkably, tumour necrosis factor-α production by RAW 264.7 murine macrophages upon challenge with lipopolysaccharide was attenuated by CETP in a dose-dependent fashion [120]. Furthermore, human CETP promoted the binding of lipopolysaccharide in vitro to both LDL and HDL [120]. Finally, the liver uptake of intravenously infused ^14^C-lipopolysaccharide from *Salmonella typhimurium* was higher in human CETP transgenic mice than in wild-type mice [120]. These findings were corroborated by a second study using the model of caecal ligation and puncture [35]. In this model, survival was higher and interleukin-6 concentrations were lower in human CETP transgenic mice than in wild-type mice, following lipopolysaccharide challenge. Furthermore, the expression of toll-like receptor 4 following lipopolysaccharide administration was lower in the liver of human CETP transgenic mice than in wild-type mice. In addition, the supplementation of macrophages from wild-type mice with recombinant human CETP decreased lipopolysaccharide uptake, toll-like receptor 4 expression, NF-κB activation, and interleukin-6 secretion [35]. Overall, the potential beneficial effects of CETP in sepsis have been attributed to an intrinsically weak binding capacity of lipopolysaccharide [121], the facilitation of the transfer of lipopolysaccharide from HDL to apolipoprotein B-containing lipoproteins [120] and enhanced clearance of lipopolysaccharide by the liver [35,120]. In the ILLUMINATE trial, when evaluating the effect of torcetrapib in patients at high risk for coronary events, an excess of deaths related to infection was observed [122,123]. However, this has not been the case with other CETP inhibitors [108]. The contrasting and striking differences in the literature [35,113,120] on the impact of CETP on sepsis may be related to species differences and the metabolic context. CETP in humans lowers HDL cholesterol and the lower HDL cholesterol concentration may represent a lower capacity to sequester lipopolysaccharide. APOE*3-Leiden.CETP mice are characterized by a lipoprotein profile that more resembles that of humans with lower levels of HDL cholesterol and higher levels of VLDL cholesterol and LDL cholesterol. Mice that only express human CETP are characterized by only a minor decline of HDL cholesterol levels since the pool of VLDL particles and LDL particles that are required for the CETP-mediated bidirectional exchange of neutral lipids is small [124]. Under these conditions, effects of CETP that are independent of transfer activity may become apparent [35,120]. The thesis is that CETP is not beneficial in the in vivo setting is corroborated by recent data of Dusuel et al. [54] who demonstrated that human CETP worsens sepsis in mice by altering the protective effects of plasma lipoproteins against endotoxemia and by increasing inflammation. These results are in direct opposition to the results of Cazita et al. [120] and Venancio et al. [35]. Overall, the evidence in favour of potential benefit of CETP inhibition in sepsis is substantial.

## 10. Conclusions and Future Perspectives

Several lines of evidence presented in this review substantiate the thesis that HDL have a profound impact on sepsis. HDL play a key role in sequestration of pathogen-associated lipids and in the reverse lipopolysaccharide transport pathway. Moreover, it may counteract the development of sepsis or mitigate the course of sepsis via its anti-inflammatory effects. Causality is strongly suggested by Mendelian randomization studies, which indicate that increased HDL cholesterol may prevent the development of sepsis and may lead to increased survival in patients with established sepsis. Causality is proven by intervention studies using apolipoprotein A-I-based therapies, CETP inhibition, or recombinant PLTP. In particular, PLTP represents a novel therapeutic target in the fight against sepsis, since this enzyme, in the context of its association with lipoproteins, plays a key role in the neutralization and clearance of endotoxins [125].

An important limitation of the current state of the art is that mechanisms that underlie changes of HDL cholesterol, of the HDL proteome, of the HDL lipidome, and of HDL-associated enzymes during sepsis are not known. Current hypotheses for the decline of HDL in sepsis are acute overconsumption of HDL particles, a decrease in liver synthesis, or a redistribution from the intravascular compartment to the extravascular compartment [79,85]. However, these proposed mechanisms are very generic and hypothetical. It is also unclear whether the alterations of HDL-associated enzymes precede changes of the HDL lipidome or are secondary to changes of the HDL lipidome. Future studies specifically comparing trauma and sepsis patients should be directed at elucidating the mechanisms that lead to the decline of HDL cholesterol in sepsis.

Notwithstanding the fact that HDL-related strategies have been successfully applied in animal models and that the rationale for these strategies is further strengthened by pathophysiological considerations and by data from observational studies and Mendelian randomization studies, several caveats should be considered. First of all, discordant results have been reported in certain domains of the field of HDL and sepsis as pointed out in the course of this review. Secondly, and most importantly, the major limitation of the field at the present is the lack of clinical translation of different HDL-targeted strategies for treatment sepsis. None of these strategies has been formally evaluated in a clinical trial in humans. Currently, no clinical trials on HDL-targeted therapies in sepsis are ongoing or registered in the European and American clinical trial databases. Recently, a case report on compassionate administration of recombinant HDL particles (rHDL, CER-001, Abionyx Pharma, Labège, France) in a patient with severe COVID-19 was published. However, this pathology is clearly distinct, compared to sepsis. Furthermore, no conclusions can be drawn based on a case report [126]. 

In general, clinical translation in the field of sepsis has been disappointing [127]. Rodents have a significantly higher resistance to sepsis than humans. The reproducibility and translational value of rodent models is a matter of debate [128] and therapies showing promise in models fail to show similar efficacy in humans [129]. This has been attributed to differences in temporal response patterns and profound differences of the innate and adaptive immune responses [129]. It has been proposed that humanised mice may provide a platform to create better sepsis models. Humanised mice are xenotransplant animals that obtain a human immune system through the expansion of human cells grafted in a mouse strain with acquired immunodeficiency. However, it is questionable whether these models really represent an advantage in terms of clinical translation. Moreover, these specific models do not address the profound differences of lipoprotein metabolism between animals and humans.

Rather than the inherent limitations of animal models, the more likely reason for poor clinical translation in the field of sepsis and the high rate of negative clinical trials is the high degree of heterogeneity in patient cohorts [130]. The classification of sepsis may be improved by the application of biological markers, which can potentiate the identification of distinct patient subclasses or endotypes [131]. This may lead to a more rational design of clinical trials and may also be beneficial in the application of HDL-targeted therapies in this field.

## Data Availability

Not applicable.
